# The Silent Infection: Cytokine Blockade, Blunted Sepsis Physiology, and Soft Tissue Infection With NLRP12-Associated Autoinflammatory Syndrome

**DOI:** 10.7759/cureus.109484

**Published:** 2026-05-23

**Authors:** Alireza Izadian Bidgoli, Alberto Gomez Veliz

**Affiliations:** 1 Internal Medicine, American University of the Caribbean School of Medicine, Cupecoy, SXM; 2 Internal Medicine, Jackson Memorial Hospital, Miami, USA

**Keywords:** blunted sepsis physiology, cytokine dysregulation, innate immune dysfunction, nlrp12-associated autoinflammatory disease, soft tissue infection

## Abstract

NLRP12-associated autoinflammatory syndrome is a rare monogenic autoinflammatory disease characterized by recurrent innate immune dysregulation and systemic inflammation. Distinguishing infection from hyperinflammatory disease activity in these patients is particularly challenging during cytokine-directed immunosuppressive therapy.

We report a 20-year-old male with NLRP12/FCAS2-associated disease, systemic juvenile idiopathic arthritis (sJIA), and prior hemophagocytic lymphohistiocytosis/macrophage activation syndrome (HLH/MAS) who presented with bilateral gluteal cellulitis, recurrent fevers, and inflammatory biomarker elevation initially concerning for sepsis. Despite progressive soft tissue findings, the patient remained hemodynamically stable without leukocytosis, lactic acidosis, or positive blood cultures. Imaging demonstrated diffuse inflammatory thickening without organized abscess formation. Multidisciplinary evaluation suggested that the patient’s recurrent fever pattern and sepsis-like physiology were driven predominantly by underlying cytokine dysregulation rather than uncontrolled systemic infection alone. Clinical improvement occurred following selective adjustment of immunosuppressive therapy in combination with antimicrobial treatment.

This case highlights the emerging concept of “immune-modified infection,” in which cytokine blockade alters the physiologic and biochemical manifestations of infection. In patients with autoinflammatory disease, inflammatory biomarker elevation may reflect immune amplification rather than microbial burden, complicating conventional sepsis assessment and management.

## Introduction

NLRP12-associated autoinflammatory disease (NLRP12-AID), also known as familial cold autoinflammatory syndrome type 2 (FCAS2), is a rare monogenic autoinflammatory disorder caused by pathogenic variants in the NLRP12 gene that result in dysregulated innate immune activation and excessive proinflammatory cytokine signaling [1]. Patients commonly present with recurrent fevers, rash, arthralgia, myalgia, and episodic systemic inflammation that can clinically resemble infectious or autoimmune conditions [1,2]. Recent advances in cytokine-directed therapies, including interleukin-1 (IL-1) blockade and Janus kinase (JAK) inhibition, have significantly improved disease control in patients with severe autoinflammatory syndromes [3]. However, these therapies may also alter the physiologic manifestations of infection, creating diagnostic uncertainty when patients present with fever and inflammatory biomarker elevation [3,4].

Sepsis is increasingly recognized as a dysregulated host response to infection rather than solely the presence of microbial invasion [5]. In patients with autoinflammatory disease receiving targeted immunomodulatory therapy, the distinction between infection-driven inflammation and sterile cytokine-mediated hyperinflammation becomes particularly challenging. Cytokine blockade may blunt traditional markers of infection such as leukocytosis, hemodynamic instability, and overt septic physiology, while persistent innate immune activation may continue to drive recurrent fever patterns and inflammatory biomarker elevation independent of uncontrolled infection [3,4]. This overlap creates a clinically important diagnostic dilemma, particularly in patients with prior episodes of macrophage activation syndrome (MAS) or hemophagocytic lymphohistiocytosis (HLH).

We present this case to emphasize the diagnostic and therapeutic challenges posed by NLRP12-AID, in which profound cytokine dysregulation may manifest as a blunted but persistent sepsis-like syndrome that confounds standard infectious workup and management.

## Case presentation

Clinical presentation

A 20-year-old male with a complex rheumatologic and autoinflammatory history, including NLRP12/FCAS2-associated cryopyrin-AID, sJIA, and prior episodes of HLH/MAS requiring chronic immunosuppressive therapy, presented with progressive bilateral gluteal pain, swelling, erythema, and drainage concerning for soft tissue infection. Several weeks prior to the current admission, he had been hospitalized for HLH/MAS characterized by markedly elevated ferritin levels and severe transaminitis, requiring escalation of immunosuppressive therapy. His treatment regimen at the time of presentation included canakinumab, anakinra, baricitinib, colchicine, and chronic prednisone therapy.

Approximately one month prior to admission, the patient first noted swelling and discomfort involving the medial gluteal folds. Symptoms progressively worsened over the following weeks with increasing tenderness and induration. Three days prior to presentation, he developed worsening bilateral buttock pain associated with subjective fevers and generalized malaise. On the day of admission, both gluteal lesions began draining spontaneously, prompting presentation to the Emergency Department for further evaluation.

He denied chest pain, dyspnea, abdominal pain, nausea, vomiting, urinary symptoms, or focal neurologic complaints. Given his profound immunocompromised state, history of severe systemic inflammatory disease, and evolving soft tissue findings, there was significant concern for superimposed infection in the setting of underlying autoinflammatory dysregulation.

Initial assessment and physical exam

On presentation, the patient appeared uncomfortable but remained hemodynamically stable and in no acute distress. Initial vital signs demonstrated a temperature of 37.6°C, heart rate of 110 beats/min, blood pressure of 108/71 mmHg, respiratory rate of 19 breaths/min, and oxygen saturation of 97% on room air. Physical examination demonstrated bilateral medial gluteal/intergluteal erythematous plaques measuring approximately 6 × 8 cm with associated firm tenderness and induration extending along the inferior gluteal folds bilaterally (Figure 1). Small superficial skin openings (1-2 mm) with scant serosanguinous drainage were present over both buttocks. The involved areas were warm to palpation without appreciable fluctuance, crepitus, bullae, or necrotic skin changes. Pain was reported as 6/10 with direct palpation and worsened with sitting and ambulation. No perianal involvement or extension into the scrotum was identified. Despite these relatively localized cutaneous findings, the patient endorsed persistent subjective fevers, generalized malaise, and progressive pain in the setting of profound immunosuppression and recent HLH/MAS, raising concern for evolving soft tissue infection versus sterile inflammatory disease activity.

**Figure 1:**
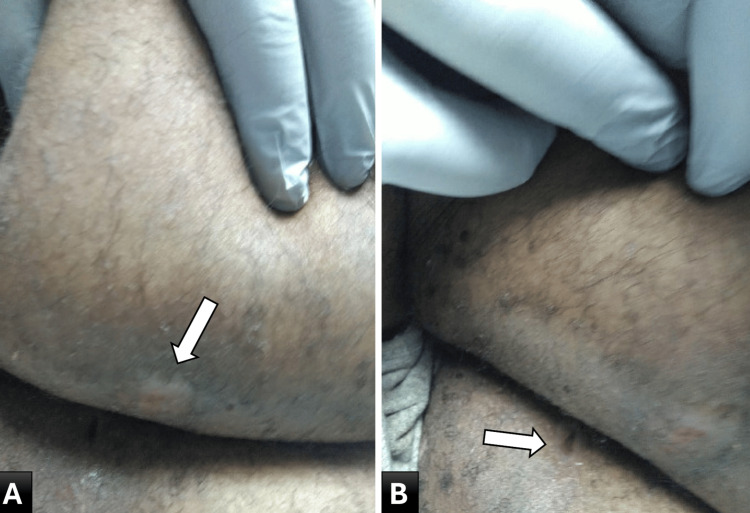
Bilateral intergluteal soft tissue lesions at presentation in NLRP12-associated autoinflammatory disease. Clinical photographs obtained on presentation demonstrating bilateral intergluteal soft tissue lesions associated with evolving cellulitis and abscess formation. Panel A demonstrates a small erythematous papular lesion with surrounding induration involving the right medial gluteal fold (white arrow). Panel B demonstrates a contralateral intergluteal draining lesion with localized erythema and superficial skin opening involving the left medial gluteal region (white arrow). Despite relatively limited superficial cutaneous findings, the patient exhibited persistent inflammatory biomarker elevation and recurrent fever episodes in the setting of NLRP12/FCAS2-associated autoinflammatory disease and chronic cytokine-directed immunomodulatory therapy.

Cardiopulmonary and abdominal examinations were otherwise unremarkable, and no active synovitis, generalized rash, or lymphadenopathy was identified. Given the patient’s history of NLRP12/FCAS2-AID with prior episodes of HLH/MAS, the initial evaluation raised concern for a complex inflammatory-infectious overlap syndrome rather than uncomplicated cellulitis alone. The relative subtlety of the cutaneous findings compared with the degree of systemic inflammatory activation created substantial diagnostic uncertainty regarding the contribution of active infection versus cytokine-driven autoinflammatory flare physiology.

Laboratory and diagnostic testing

Initial laboratory evaluation demonstrated preserved hematologic and metabolic stability despite ongoing inflammatory symptoms. White blood cell count remained within normal limits at 9 × 10³/µL with relative monocytosis and lymphocytosis. Serum lactate was normal at 2.2 mmol/L despite recurrent fever episodes and concern for soft tissue infection. Liver-associated enzymes were elevated, including aspartate aminotransferase (AST) 86 U/L and alanine aminotransferase (ALT) 169 U/L, with total bilirubin elevated to 2.1 mg/dL. Blood cultures remained negative throughout hospitalization. These findings created diagnostic uncertainty regarding whether the patient’s inflammatory physiology reflected evolving infection, cytokine-driven autoinflammatory activity, or overlap between both processes. Table 1 summarizes the laboratory findings during admission, during hospitalization, and at discharge. 

**Table 1:** Laboratory findings during hospitalization in a patient with NLRP12-associated autoinflammatory disease and gluteal soft tissue infection. Serial laboratory findings obtained during hospitalization demonstrating persistent systemic inflammatory activation despite a relatively localized soft tissue infectious burden. The patient exhibited marked hyperferritinemia, elevated lactate dehydrogenase, transaminitis, hypertriglyceridemia, and fluctuating neutrophil counts consistent with ongoing innate immune dysregulation and macrophage activation physiology. Notably, inflammatory biomarker elevation appeared disproportionate to the degree of radiographic and microbiologic evidence of infection, supporting the concept of cytokine-driven inflammatory amplification in NLRP12-AID. Abbreviations: AST: aspartate aminotransferase; ALT: alanine aminotransferase; LDH: lactate dehydrogenase; CRP: C-reactive protein; SGOT: serum glutamic-oxaloacetic transaminase; SGPT: serum glutamic-pyruvic transaminase

Laboratory Parameter	Admission	Range During Hospitalization	Discharge	Reference Range (Unit)
White blood cell count	9.0	3.9-5.3	4.5	4.5-11.0 × 10³/µL
Hemoglobin	12.1	11.5-11.8	11.6	13.5-17.5 g/dL
Platelet count	186	170-244	198	150-400 × 10³/µL
Absolute neutrophil count	7.9	1.1-2.0	1.1	1.8-7.7 × 10³/µL
Absolute lymphocyte count	0.5	0.5-1.9	1.9	1.0-4.8 × 10³/µL
Sodium	136	132-135	135	136-145 mmol/L
Creatinine	0.51	0.50-0.94	0.61	0.7-1.3 mg/dL
AST (SGOT)	86	69-151	82	10-40 U/L
ALT (SGPT)	169	156-190	171	7-56 U/L
Total bilirubin	2.1	1.2-1.5	1.2	0.2-1.2 mg/dL
Albumin	3.5	3.1-3.5	3.5	3.5-5.0 g/dL
Ferritin	6,020	9,170-10,000	9,170	24-336 ng/mL
LDH	848	669-1,276	669	140-280 U/L
Triglycerides	204	163-235	163	<150 mg/dL
C-reactive protein	-	3.2-5.4	3.2	<0.5 mg/dL
Lactic acid	2.2	1.5-2.2	1.5	0.5-2.0 mmol/L
Fibrinogen	287	273-348	309	200-400 mg/dL

Computed tomography (CT) of the pelvis with intravenous contrast demonstrated ill-defined soft tissue thickening involving the bilateral inferior intergluteal folds with surrounding subcutaneous stranding, greater on the left side, without organized fluid collection or drainable abscess (Figure 2). The radiographic findings were interpreted as suspicious for cellulitis without overt deep space infection, necrotizing soft tissue involvement, or osteomyelitis. The relatively limited radiographic burden contrasted with the patient’s persistent systemic inflammatory manifestations and recurrent fever physiology.

**Figure 2:**
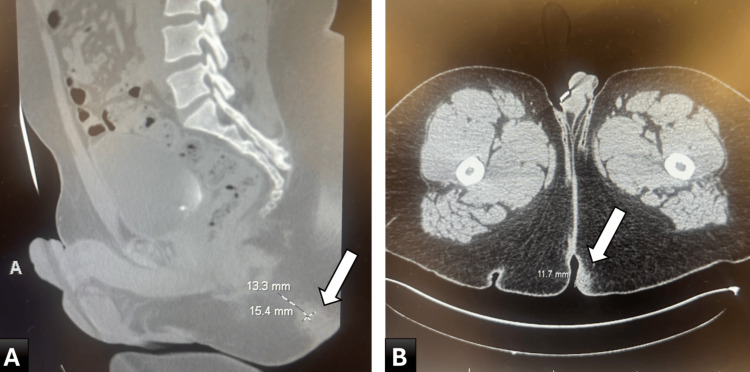
Computed tomography demonstrating localized intergluteal inflammatory soft tissue thickening without organized abscess formation. Contrast-enhanced computed tomography of the pelvis obtained at admission demonstrating focal inflammatory soft tissue thickening involving the inferior intergluteal region. Panel A (sagittal view) demonstrates localized superficial soft tissue thickening within the intergluteal fold (white arrow). Panel B (axial view) demonstrates bilateral medial gluteal inflammatory change with surrounding subcutaneous stranding and focal soft tissue involvement adjacent to the intergluteal cleft (white arrow). No organized fluid collection, deep fascial extension, osteomyelitis, or drainable abscess was identified. The relatively limited radiographic burden despite recurrent fever episodes and persistent systemic inflammatory activation highlights the dissociation between imaging severity and inflammatory physiology in cytokine-modulated NLRP12-associated autoinflammatory disease.

Additional imaging during the broader hospitalization course further illustrated the complexity of interpreting inflammatory symptoms in this patient. Chest radiography obtained during a prior febrile admission demonstrated mild left basilar subsegmental atelectatic change without focal consolidation or pulmonary edema (Figure 3A). Repeat chest radiography during the current hospitalization demonstrated low lung volumes with vascular crowding and mild bilateral interstitial opacities without focal bacterial pneumonia or other definitive infectious source (Figure 3B). Collectively, the imaging findings across multiple organ systems remained relatively nonspecific despite recurrent inflammatory episodes, supporting the possibility that dysregulated innate immune activation substantially contributed to the patient’s recurrent “sepsis-like” presentations.

**Figure 3:**
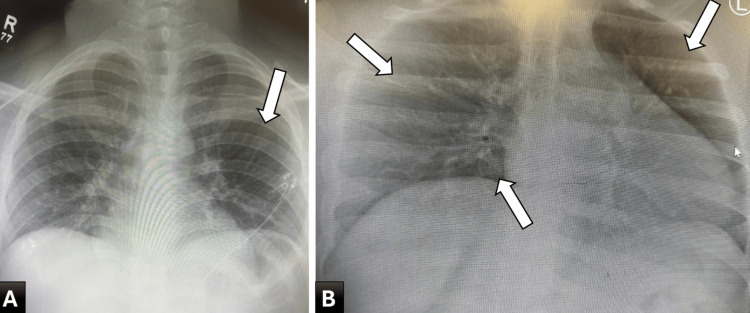
Serial chest radiographs demonstrating progression of pulmonary inflammatory findings during hospitalization. Panel A (old radiography) demonstrates the initial chest radiograph obtained at presentation showing mild left basilar subsegmental atelectatic change without focal pulmonary consolidation (white arrow). Panel B (new radiography) demonstrates follow-up chest radiography obtained later during hospitalization revealing interval development of diffuse bilateral interstitial pulmonary opacities with low lung volumes and bronchovascular crowding (white arrows), without focal lobar consolidation or pleural effusion. These findings were interpreted as nonspecific inflammatory pulmonary changes and were disproportionate to the relatively localized soft tissue infectious burden identified on pelvic imaging, further supporting a systemic inflammatory phenotype in the setting of cytokine dysregulation and immune modulation.

Multidisciplinary board discussion

Given the patient’s extensive history of systemic autoinflammatory disease and ongoing immunosuppression, multiple specialty teams were involved early in the hospitalization, including Rheumatology, Infectious Disease, and the primary Internal Medicine service. Rheumatology evaluated the patient in the setting of known NLRP12/FCAS2-AID with prior HLH/MAS and recognized the diagnostic challenge of differentiating infectious symptoms from inflammatory disease flare. The rheumatology team emphasized the patient’s recent severe inflammatory episodes and discussed the balance between maintaining immunosuppressive therapy to prevent recurrence of HLH/MAS while simultaneously reducing immunosuppression in the setting of active infection.

The Infectious Disease service reviewed the patient’s presentation and imaging findings and agreed that the clinical picture was most consistent with localized skin and soft tissue infection involving the intergluteal region. Their assessment noted concern for cellulitis progressing toward abscess formation despite initially negative imaging for drainable collections. In discussion with Rheumatology, a decision was made to continue selected anti-inflammatory therapies while temporarily holding baricitinib during active infection because of concern for further immunosuppression. Daily monitoring of inflammatory markers, liver function tests, and blood counts was recommended because of the patient’s prior history of MAS/HLH and severe systemic inflammatory decompensation.

Diagnosis and management

The patient was admitted for management of suspected bilateral gluteal cellulitis and soft tissue infection in the setting of profound immunosuppression from chronic treatment with multiple immunomodulatory agents, including IL-1 inhibition, JAK inhibition, and corticosteroid therapy. Initial evaluation demonstrated persistent systemic inflammatory symptoms with bilateral gluteal erythema, induration, tenderness, and spontaneous drainage concerning for evolving soft tissue infection versus autoinflammatory disease flare. Empiric broad-spectrum intravenous antimicrobial therapy with vancomycin, cefepime, and metronidazole was initiated on admission, and both Infectious Disease and Acute Care Surgery services were consulted for multidisciplinary evaluation and management.

Blood cultures obtained during hospitalization remained negative. Despite initial CT imaging demonstrating no drainable fluid collection, persistent focal tenderness, induration, and drainage prompted surgical reassessment. Bedside incision and drainage of a left gluteal abscess was subsequently performed with evacuation of purulent material and placement of wet-to-dry packing for ongoing local wound care. Following source control, the patient demonstrated gradual improvement in pain, drainage, and local inflammatory findings.

Given the absence of hemodynamic instability, negative blood cultures, lack of progressive necrosis, and improving local examination after drainage, antimicrobial therapy was subsequently narrowed in consultation with Infectious Disease specialists. Cefepime and metronidazole were transitioned to ceftriaxone-based therapy with continued anaerobic coverage due to the intergluteal location of infection and concern for polymicrobial soft tissue involvement. The patient completed an approximately seven-day total antibiotic course with continued daily wound care and serial reassessment by surgery, infectious disease, rheumatology, and internal medicine teams.

Throughout hospitalization, management remained challenging because persistent fevers and markedly elevated inflammatory markers raised concern for concurrent cytokine-driven autoinflammatory activity in addition to infection. Rheumatology closely monitored ferritin levels, liver enzymes, inflammatory markers, and clinical status, given the patient’s recent history of HLH/MAS. Immunosuppressive therapy was carefully adjusted during admission, including temporary modification of baricitinib therapy, continuation of corticosteroids, and ongoing IL-1 inhibition management. As the patient’s gluteal findings improved and no evidence of progressive infection emerged, persistent intermittent fevers were ultimately felt to be more consistent with underlying inflammatory disease activity rather than uncontrolled bacterial infection.

By the time of discharge planning, the patient was afebrile with improving wound appearance, decreased tenderness, stable hemodynamics, and no further need for surgical intervention. He was discharged with continuation of antimicrobial therapy to complete the planned treatment course, ongoing wound care instructions, and close outpatient follow-up with rheumatology and surgical services.

Clinical outcome

During hospitalization, the patient remained hemodynamically stable without evidence of systemic sepsis or recurrent HLH/MAS flare. Serial examinations demonstrated gradual improvement in gluteal erythema, induration, and drainage following initiation of intravenous antibiotics and bedside drainage procedures. Blood cultures remained negative, and no deeper pelvic extension of infection was identified. Although liver function abnormalities persisted, transaminase levels showed partial improvement compared with prior documented inflammatory episodes.

The patient tolerated antimicrobial therapy without major complications and did not develop worsening systemic inflammatory manifestations during admission. Multidisciplinary coordination between Internal Medicine, Rheumatology, and Infectious Disease allowed for careful balancing of infection control and management of his underlying autoinflammatory disease. He was ultimately discharged with ongoing outpatient rheumatologic follow-up, continuation of tailored immunosuppressive therapy, and close monitoring for recurrent infection or inflammatory flare.

## Discussion

Background 

NLRP12-AID, also known as FCAS2, is a rare monogenic autoinflammatory disorder caused by pathogenic variants in the NLRP12 gene and inherited primarily in an autosomal dominant pattern [1,6]. NLRP12 functions as an intracellular regulator of innate immune signaling through modulation of inflammasome activity and suppression of nuclear factor-kappa B (NF-κB)-mediated inflammatory pathways [6,7]. Dysregulation of this pathway results in recurrent sterile inflammatory activation characterized by episodic fever and multisystem inflammatory manifestations [1,6,7].

The disease was initially described in association with hereditary periodic fever syndromes and cold-induced inflammatory attacks, although subsequent studies have demonstrated substantial phenotypic heterogeneity extending beyond classic cold-triggered disease [6,7]. Because of the rarity of the condition and variability in clinical expression, the true prevalence of NLRP12-AID remains unknown and is likely underestimated [1,3]. Clinical manifestations often begin during childhood, but delayed diagnosis into adulthood is increasingly recognized because of overlap with infectious, rheumatologic, and autoimmune conditions [1,3]. Common features include recurrent fever, urticaria-like rash, arthralgia, myalgia, abdominal pain, lymphadenopathy, and persistent inflammatory biomarker elevation during disease flares [1,3,7].

Environmental triggers, including cold exposure, physiologic stress, and intercurrent infection, may precipitate inflammatory episodes in genetically susceptible individuals [6,7]. Increasing use of cytokine-directed therapies, including IL-1 blockade and JAK inhibition, has improved disease control in severe autoinflammatory syndromes but may simultaneously alter the physiologic manifestations of infection and systemic inflammation [3,7]. Consequently, patients may develop recurrent fever patterns and sepsis-like inflammatory physiology despite minimal microbiologic evidence of uncontrolled infection, creating substantial diagnostic and therapeutic uncertainty [3,7].

Pathogenesis/pathophysiology

NLRP12-AID is driven by dysregulation of the innate immune system rather than infection, autoantibody formation, or malignant transformation [8,9]. NLRP12 normally functions as a negative regulator of inflammatory signaling pathways, particularly NF-κB and inflammasome-mediated cytokine activation [8,10]. Pathogenic variants impair this regulatory function, resulting in excessive production of proinflammatory cytokines and recurrent episodes of sterile systemic inflammation [8,10]. Monocytes, macrophages, and neutrophils play a central role in this inflammatory cascade through exaggerated innate immune activation and cytokine release [9,10].

Clinically, this dysregulated inflammatory state manifests as recurrent fever, rash, arthralgia, myalgia, lymphadenopathy, elevated acute-phase reactants, and episodic hyperinflammatory syndromes including MAS [8-10]. Environmental stressors, cold exposure, and intercurrent infection may amplify cytokine activation and precipitate disease flares [8,9]. Importantly, cytokine-directed therapies such as IL-1 blockade and JAK inhibition may suppress conventional physiologic markers of infection while persistent innate immune activation continues to drive fever and inflammatory biomarker elevation [9,11]. This altered immune state may create a “silent sepsis” phenotype, in which inflammatory manifestations appear disproportionate to the degree of microbiologic infection.

Comparative analysis of this case with the current literature 

Clinical Presentation

NLRP12-AID is a clinically heterogeneous disorder characterized by recurrent fever, episodic systemic inflammation, urticaria-like rash, arthralgia, myalgia, abdominal pain, and variable multisystem involvement resulting from dysregulated innate immune signaling [8,10,12]. Delayed diagnosis into adulthood is increasingly recognized because clinical manifestations frequently overlap with infection, autoimmune disease, and systemic inflammatory syndromes [10,12]. Similar to previously reported cohorts, our patient demonstrated recurrent inflammatory episodes, persistently elevated inflammatory biomarkers, and prior hyperinflammatory complications, including HLH/MAS [8,10,12,13]. However, the distinguishing feature of this case was the development of a clinically attenuated yet biologically amplified sepsis-like phenotype during chronic cytokine-directed immunotherapy [8,11,13].

Despite progressive gluteal cellulitis requiring incision and drainage and broad-spectrum antimicrobial therapy, the patient lacked many conventional markers of severe bacterial soft tissue infection, including marked leukocytosis, severe lactic acidosis, or hemodynamic instability [8,11,13]. This dissociation between relatively localized infectious findings and disproportionate systemic inflammatory activation has not been extensively emphasized in prior NLRP12-AID reports [8,10,12]. Existing literature suggests that IL-1 blockade and JAK inhibition may blunt classical inflammatory responses during infection while incompletely suppressing underlying innate immune dysregulation [8,11,13,14]. Our case further expands this concept by illustrating how recurrent fever and inflammatory biomarker elevation may reflect maladaptive cytokine amplification disproportionate to the severity of infection itself [8,11,13,14].

Diagnostic Workup

Diagnosis of NLRP12-AID remains particularly challenging because recurrent febrile episodes frequently mimic occult infection, autoimmune disease, or systemic inflammatory syndromes [10,12]. Published series emphasize that many patients undergo extensive infectious and rheumatologic evaluations before recognition of an underlying monogenic autoinflammatory disorder [10,12]. In our patient, this diagnostic uncertainty was reflected by persistent fevers, markedly elevated inflammatory markers, and recurrent hyperferritinemia despite relatively localized gluteal soft tissue findings and negative blood cultures, creating difficulty distinguishing uncontrolled infection from cytokine-driven autoinflammatory activity. Similar diagnostic ambiguity has been described in patients with inflammasomopathies and cytokine dysregulation syndromes, particularly in those with prior HLH/MAS and chronic immunomodulatory therapy [8,11-13].

Radiographic findings in this case were also atypical compared with conventional bacterial soft tissue infection. CT demonstrated diffuse inflammatory thickening and subcutaneous stranding without early organized abscess formation despite progressive symptoms [11,13,14]. Subsequent bedside incision and drainage with evacuation of purulent material ultimately confirmed a true infectious component despite initially limited imaging findings. This discordance between relatively modest radiographic burden and disproportionate systemic inflammatory activation raises the possibility that cytokine-directed therapy alters tissue-level inflammatory compartmentalization, neutrophil recruitment, macrophage signaling, and abscess maturation [8,11,13,14].

Existing literature describing cytokine blockade in autoinflammatory disease suggests that targeted immunomodulation may obscure conventional markers of infection while simultaneously failing to fully suppress underlying innate immune dysregulation [8,11,13,14]. Consequently, the principal diagnostic challenge in this case was not merely identifying infection, but distinguishing infection-driven inflammation from cytokine-amplified autoinflammatory activity in a patient predisposed to HLH/MAS [8,11-14].

Management

Current management of NLRP12-AID focuses primarily on suppression of dysregulated innate immune signaling through corticosteroids, colchicine, IL-1 blockade, and targeted biologic therapies [8,10,14]. However, evidence-based guidance regarding management of active infection in these patients remains limited because available literature consists predominantly of observational cohorts and isolated case reports [8,10,12]. Published experience suggests that abrupt withdrawal of immunomodulatory therapy in patients with severe autoinflammatory disease may precipitate rebound hyperinflammation, recurrent MAS, or cytokine storm syndromes [11,13,14].

Management of our patient reflected this emerging precision-based immunologic strategy. Broad-spectrum antimicrobial therapy was initiated because of concern for cellulitis in an immunocompromised host, while immunosuppressive therapy was selectively modified rather than universally discontinued [11,13,14]. Temporary interruption of baricitinib with continuation of selected anti-inflammatory agents allowed simultaneous control of infection risk and prevention of uncontrolled inflammasome rebound [8,11-14]. Compared with traditional infectious disease paradigms, this approach emphasizes dynamic immunologic balancing rather than binary withdrawal or continuation of immunosuppression [11,13,14]. Our case, therefore, supports the evolving concept that immune modulation itself may represent an essential component of infectious management in patients with monogenic autoinflammatory disease [8,11,13,14].

Outcome and Follow-Up

Reported outcomes in NLRP12-AID vary according to disease severity, frequency of inflammatory flares, and development of systemic complications such as MAS or HLH [8,10,12]. Recent studies demonstrate improved inflammatory control and reduced disease burden with cytokine-directed therapies, although recurrent inflammatory episodes and infection-related complications remain common [8,14]. In our patient, combined antimicrobial therapy, selective immunomodulatory adjustment, and procedural drainage resulted in gradual clinical improvement without progression to septic shock or recurrent HLH/MAS [11,13,14].

Importantly, the patient’s clinical trajectory appeared more consistent with controlled localized infection occurring within a persistently dysregulated inflammatory environment rather than uncontrolled disseminated sepsis alone [8,11,13,14]. This observation reinforces the concept that inflammatory biomarkers, recurrent fever patterns, and persistent hepatic dysfunction in NLRP12-AID may not directly correlate with microbial burden [8,11-14]. Longitudinal follow-up, therefore, remains essential because recurrent inflammatory activation, secondary infection, and hyperinflammatory relapse may occur despite apparent clinical stability [8,10-14].

Literature Review

Recent literature increasingly recognizes monogenic autoinflammatory diseases as disorders of immune dysregulation in which infection and inflammation become biologically intertwined rather than clinically separable processes [8,10-14]. However, few reports specifically examine how cytokine blockade may fundamentally alter the physiologic phenotype of infection itself [8,11,13,14]. Our case contributes to the emerging concept of “immune-modified infection,” in which targeted immunotherapy and genetically altered innate immune signaling reshape the biochemical, radiographic, and systemic manifestations of sepsis [8,11,13,14]. Compared with previously published reports, this case uniquely highlights the dissociation between inflammatory physiology and microbiologic burden in a patient with NLRP12-associated disease receiving chronic cytokine-directed therapy [8,11-14]. As biologic immunomodulators become increasingly prevalent across rheumatologic, dermatologic, oncologic, and inflammatory diseases, recognition of this altered infectious phenotype may become increasingly important for future diagnostic interpretation, longitudinal surveillance, and individualized immunologic management [8,11,13,14].

What we learned from this case 

This case highlights several important clinical challenges in the management of monogenic autoinflammatory disease complicated by infection. First, distinguishing active infection from cytokine-driven inflammatory flare can be exceptionally difficult in patients with NLRP12-AID, particularly in the setting of persistent fevers, hyperferritinemia, and prior HLH/MAS. In our patient, localized gluteal soft tissue infection coexisted with disproportionate systemic inflammatory activation, creating substantial diagnostic uncertainty regarding the relative contribution of uncontrolled infection versus autoinflammatory amplification.

Second, chronic cytokine-directed immunomodulatory therapy may blunt conventional physiologic and biochemical markers of sepsis. Despite clinically progressive soft tissue infection requiring incision and drainage and broad-spectrum antimicrobial therapy, the patient lacked marked leukocytosis, severe lactic acidosis, hemodynamic instability, or extensive radiographic findings typically associated with severe bacterial infection. This case suggests that IL-1 and JAK pathway inhibition may alter how infection manifests clinically in patients with dysregulated innate immunity.

Finally, management required an individualized multidisciplinary balancing strategy integrating antimicrobial therapy, procedural source control, and careful adjustment of immunosuppressive medications. Excessive reduction of immunosuppression carried risk for recurrent HLH/MAS and hyperinflammatory rebound, whereas inadequate infectious management risked progression of soft tissue infection. This case, therefore, emphasizes the importance of interpreting infection within the context of the patient’s underlying immune architecture rather than relying solely on conventional sepsis paradigms.

## Conclusions

This case illustrates how targeted cytokine-directed immunotherapy can fundamentally alter the clinical expression of infection in patients with monogenic autoinflammatory disease. In NLRP12-AID, recurrent fever, inflammatory biomarker elevation, and sepsis-like physiology may reflect dysregulated innate immune amplification rather than uncontrolled microbial burden alone. Our patient developed progressive soft tissue infection despite blunted conventional markers of sepsis, emphasizing how cytokine blockade may modify the physiologic, biochemical, and radiographic manifestations of infection.

This case further underscores the importance of distinguishing infection-driven inflammation from hyperinflammatory disease activity, particularly in patients with prior HLH/MAS who remain vulnerable to catastrophic inflammatory rebound. Recognition of this altered infectious phenotype is critical for accurate diagnostic interpretation, individualized immunomodulatory management, and avoidance of both undertreatment of infection and excessive withdrawal of immunosuppressive therapy. As targeted biologic therapies become increasingly prevalent, clinicians should recognize that chronic cytokine modulation may alter infection presentation, requiring integration of genetic immune phenotype, current immunosuppression, and infectious evidence when interpreting inflammatory biomarkers.
